# The emission of γ-Ray beams with orbital angular momentum in laser-driven micro-channel plasma target

**DOI:** 10.1038/s41598-019-55217-4

**Published:** 2019-12-11

**Authors:** B. Feng, C. Y. Qin, X. S. Geng, Q. Yu, W. Q. Wang, Y. T. Wu, X. Yan, L. L. Ji, B. F. Shen

**Affiliations:** 1 State Key Laboratory of High Field Laser Physics, Shanghai Institute of Optics and Fine Mechanics, Chinese Academy of Sciences, Shanghai, 201800 China; 2 University of Chinese Academy of Sciences, Beijing, 100049 China; 3 Shanghai Normal University, Shanghai, 200234 China; 4 Center for Excellence in Ultra-intense Laser Science, Chinese Academy of Sciences, Shanghai, 201800 China

**Keywords:** Plasma-based accelerators, Laser-produced plasmas

## Abstract

We investigated the emission of multi-MeV γ-Ray beams with orbital angular momentum (OAM) from the interaction of an intense circularly polarized (CP) laser with a micro-channel plasma target. The driving laser can generate high energy electrons via direct laser acceleration within the channel. By attaching a plasma foil as the reflecting mirror, the CP laser is reflected and automatically colliding with the electrons. High energy gamma-photons are emitted through inverse Compton scattering (ICS) during collision. Three-dimensional particle-in-cell simulations reveal that the spin angular momentum (SAM) of the CP laser can be transferred to the OAM of accelerated electrons and further to the emitted gamma-ray beam. These results may guide future experiments in laser-driven gamma-ray sources using micro-structures.

## Introduction

Owing to the rapid progress in laser technology, ultra-relativistic femtosecond laser beams with high pulse contrast have become available. The newly developed laser-pulse cleaning technique has achieved a contrast better than 10^10^ at the nanosecond level^1^. These advancements have motivated the use of high contrast laser to interact with plasma targets with fine structures, for the purpose of increasing laser absorption and the subsequent energy conversion in the secondary radiation^2–6^, as well as ion acceleration^7,8^. For example, nanowire-array targets^9,10^ and nanoparticles^11^ have been proposed to enhance laser absorption theoretically. Nano-structured target have been realized to increase electron^12–14^ and proton energies^15^ and X-ray generation^16^ in previous experiments. Recently, micro-channel plasma (MCP) targets have shown novel effects on light intensification^12^, proton acceleration^17^, x/gamma-ray generation^18^ and even electron-positron pair production^19^, in various simulations. The first experiment of laser-driven MCP target by Snyder *et al*. showed enhanced acceleration and efficient guiding of high energy electrons^20^. These result from the direct-laser-acceleration within the channel and the self-generated electric-magnetic fields of the structure. Success of the experiment suggests that laser-driven micro-structures are promising in producing a unique source of electrons, which can support sequential applications like proton acceleration and secondary radiations.

According to previous research^21^, a conversion from SAM to OAM based on high-order harmonic generation was achieved when an intense CP laser interacting with a plane foil. Here we show that the micro-channel structure can be utilized to generate high energy (>1 MeV) gamma-ray photons carrying the orbital angular momentum (OAM), via particle-in-cell (PIC) simulations. Through single-pulse laser-electron collision^22–24^ within the channel, a circularly-polarized (CP) laser can transfer its spin angular momentum (SAM) to electrons. Then the latter would emit multi-MeV *γ*-Ray beam with OAM from the interaction with the driving laser based on inverse Compton scattering. Methods of generating photons carrying OAM is proposed previously, via the interaction of the CP Laguerre-Gaussian (LG) laser with a plane solid target^25^ or a counter-propagating ultrarelativistic electron beam^26^, where the OAM of high-energy gamma-ray photons is transferred from both the SAM and OAM of the driving laser. By using an ordinary CP laser with a MCP target, we point out that electron bunches moving with the CP laser obtain the OAM transferred from the SAM of the CP laser, as it does in near-critical-density plasma^27^. These electrons transfer the OAM to high-energy photons when ICS is triggered. We also revealed that about half of the OAM of the gamma photons comes from the electrons and half from the scattering laser. The use of a CP laser pulse instead of LG laser is well-suited for future experiments. The alignment between the drive laser and the channel target can be mitigated by using of a compact array of many identical units of channels, which has been discussed in a previous study in terms of laser-electron acceleration^12^.

## Results and Analysis

**Figure 1:**
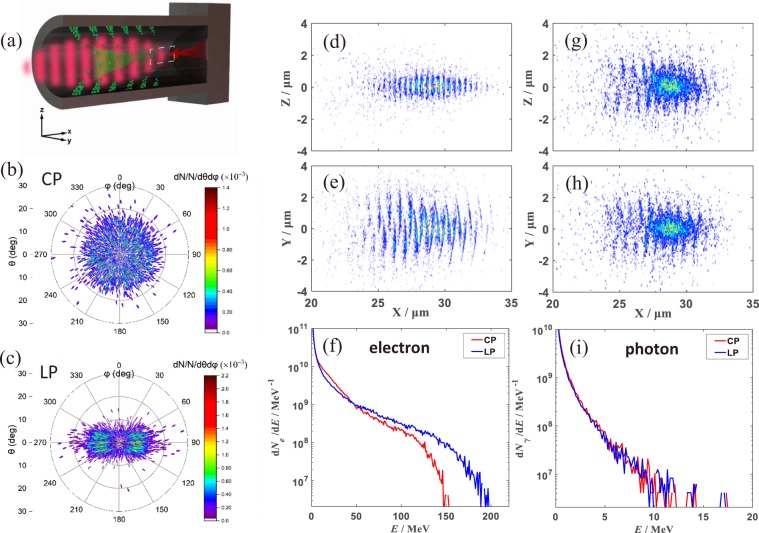
(**a**) Three-dimensional PIC simulations of an intense short-pulse laser interacting with a MCP target: the laser pulse (I = 10^21^ W/cm^2^) propagates along the x direction from right to left (green balls and periodic red blocks represent electrons and the incident laser, respectively. Green and red arrows indicate the direction of movement of electrons and the laser.). The MCP target consists of a channel of 15 μm long and 6 μm diameter attached to 5-μm-thick substrate. The angular distribution of photons in the case of (**b**) the CP laser and (**c**) the LP laser. (**d**,**e**) and (**g**,**h**) are the density distributions of photons in the case of the LP laser and the CP laser at 125 fs ((**d**,**g**) on the x-z plane, (**e**,**h**) on the x-y plane); the energy spectra of electrons (**f**) and photons (**i**) at 110 fs and 130 fs, in the case of the CP laser (red line) and the LP laser (blue line).

As discussed in previous work, when a laser beam radiates on a MCP target, electrons on the surface of channel will be extracted into the laser field and accelerated via direct laser acceleration (DLA)^13,28,29^. Electrons are periodically separated by one laser wavelength. And then the electrons in appropriate phases co-propagate with the driving laser obtaining high energy, as sketched in Fig. 1(a). When the laser pulse approaches the flat foil on the rear side of the channel, these electron bunches collide with the laser pulse reflected from the substrate and simultaneously triggered the ICS process, resulting in high energy *γ* photon emission. On the laser-polarization plane (y-z plane), we find that the density peaks of gamma-photons are separated by one laser wavelength on each side of the y-axis, with a π/2 phase shift. Both groups of electrons overlap when viewed on the x-z plane, showing a half-laser-wavelength period, as shown in Fig. 1(d,e). The distinctive distributions are consistent with the asymmetric field structure of the LP laser (field polarization direction). However, the intervals of photon density peaks are both one laser wavelength on either side for CP laser as shown in Fig. 1(g,h), a natural result from the symmetric polarization of the incident laser beam.

The energy spectra of electrons in the case of CP (red line) and LP (blue line) can be seen in Fig. 1(f). The cut off energy of electrons (at 110 fs) are about 150 MeV for the case of CP and 200 MeV for the case of LP. In both cases, one sees similar spectrum for the emitted gamma photons in Fig. 1(i), with cut-off energy beyond 10 MeV. Considering the electron energy and the laser amplitude, the collision between them is well in the non-linear Compton Scattering regime. The discrete photon emission process is characterized by the invariant parameter $\eta =(\gamma /{E}_{Sch})|{{\boldsymbol{E}}}_{\perp }+{\boldsymbol{\upsilon }}\times {\boldsymbol{B}}|$^30,31^. This is also often referred to as the QED parameter that measures the photon energy over the energy of the emitting electron. Here *γ* is the Lorentz factor of the electrons, ${{\rm{E}}}_{\perp }$ is the electric field perpendicular to the electron velocity, B is the laser magnetic field and ${E}_{Sch}={m}_{e}^{2}{c}^{3}/e\hslash \approx 1.3\times {10}^{18}V{m}^{-1}$ is the Schwinger-limit electric field^32^ ($\hslash$ is the reduced Planck constant). For electrons co-propagating with the laser pulse, the electric force is balanced by the magnetic force, resulting in ${\rm{\eta }}\to 0$, which is unfavorable for efficient high-energy radiation. However, when being reflected by the substrate, the laser beam head-on collides with the electron beam, leading to a QED parameter of $\eta \approx 2\gamma |{E}_{\perp }|/{E}_{Sch}$. Based on the electron energy spectra in the channel, we have $\eta \,\approx \,$0.148 for LP laser. The highest energy of the emitted photons can be estimated to ${\rm{h}}\nu =0.44\eta \gamma {m}_{e}{c}^{2}\approx 13\,$MeV^33^, which is in good agreement with the simulation in Fig. 1(i). After the collision between electrons and the laser pulse, 2.5 × 10^11^ gamma-photons are emitted and the energy conversion efficiency from the laser pulse to gamma-photons can reach to 1.8‰. The laser-MCP interaction is particularly attractive in generating high flux broadband gamma-photons at moderate photon energies, due to the enormous electron charge (about 27nC at > 10 MeV) from overdense plasma structures.

Figure 1(b) exhibit the angular distribution of the gamma-photons (>2 MeV) from ICS in case of the CP laser, where *θ* denotes the polar angle of the photons momentum with respect to x axis and *φ* is the azimuthal angle between the projection of the photons momentum into yz-plane and z axis, respectively. Most of energetic photons are predominantly distributed within an emission polar angle θ < 20°. In the case of LP laser, most energetic photons are distributed in the vicinity of φ = 90° and φ = 270°, which is attributed to the LP laser polarization, as shown in Fig. 1(c).

**Figure 2:**
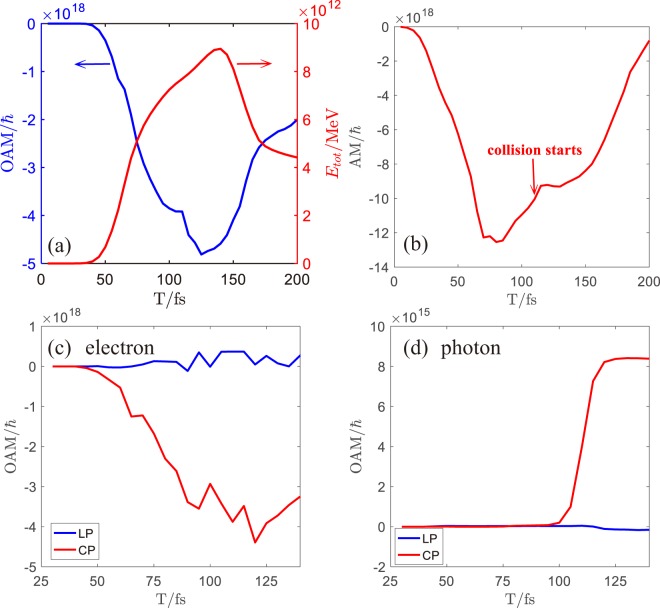
Time evolution of (**a**) the OAM of the electrons (blue solid line) and total electron energy (red solid line) in long channel (35μm), (**b**) the AM of the laser in short channel, (**c**) the OAM of the electrons and (**d**) photons in the case of the LP laser and the CP laser in short channel (15μm).

A very interesting feature of the CP laser pulse is that it can transfer its spin angular momentum (SAM) efficiently to the orbital angular momentum (OAM) of the accelerated electrons. To see this through, we use a sufficiently long channel (35μm) and remove the flat foil. The time evolution of the OAM for all the electrons (blue solid line) is shown in Fig. 2(a) together with the total electron energy (red solid line). Here, the electron/photons OAM is calculated by ${\rm{OAM}}={{\rm{yp}}}_{{\rm{z}}}-{{\rm{zp}}}_{{\rm{y}}}$, where py, = p_z_ are the momentum of particles in the y and z directions, respectively. The OAM of electrons increases continuously and peaks at 125 fs (the minus sign of the OAM comes from the direction of the reference axis taken for statistics). The electrons gradually lose their OAM. This behavior is synchronized with the energy evolution. The electrons first gain energy from the laser field and then lose it, exhibiting a peak at 125 fs. This interaction moment denotes the start of the de-phasing stage, where deceleration and OAM-loss happen. Therefore, to maximize the gain of energy and OAM for electrons, we choose to a channel length of 15μm (corresponding to about 125 fs propagation time before collision) and place the reflecting foil by it.

With the optimized channel length and reflecting foil, the total angular momentum (AM) of the CP laser is shown in Fig. 2(b). The total AM of the laser is calculated by AM = ***r*** × *ε*_0_**(*E*** × ***B***). We can find that the total AM of the CP laser first increases as the laser beam enters the simulation box. Simultaneously, the laser begins to transfer its AM to the electrons at about 40 fs. When the laser pulse is fully in the simulation window, the total AM starts to decline at 70 fs, while electrons are gaining OAM efficiently, as seen in Fig. 2(c). The collision takes place at about 110 fs, leading to a rapid boost in the gamma-photon OAM due to the ICS process, as illustrated in Fig. 2(d). The electron OAM is disturbed by the collision and then decline later on. The above effect is not active when we switch the driving laser to be linearly polarized. We see that neither electrons nor gamma-photons carry any OAM during the whole interaction. The reason is apparent: LP lasers do not contain any SAM.

Before collision, electrons accelerated by the CP laser within the channel carry maximum OAM of 4.27 × 10^18^ *ħ*, about 34% of that for the driving beam. The photons gain, after collision, OAM of 8.08 × 10^15^ *ħ*, reaching an efficiency of 10^−3^ from the CP laser. In addition, the average OAM of a single electron (>10 MeV) is up to 1.56 × 10^7^ *ħ* and according to ICS a single photon (>0.6 MeV, calculated based on the 10 MeV electron energy) gains averaged OAM of 2.3 × 10^5^ *ħ*, suggesting a conversion efficiency of 1.5% for OAM. While the plasma may act as a non-trivial background affecting the ICS rates^34^, we found that in our case the plasma is opaque to the laser field and the latter is dominating in the head-on collision geometry.

## Transfer Mechanism of OAM

**Figure 3:**
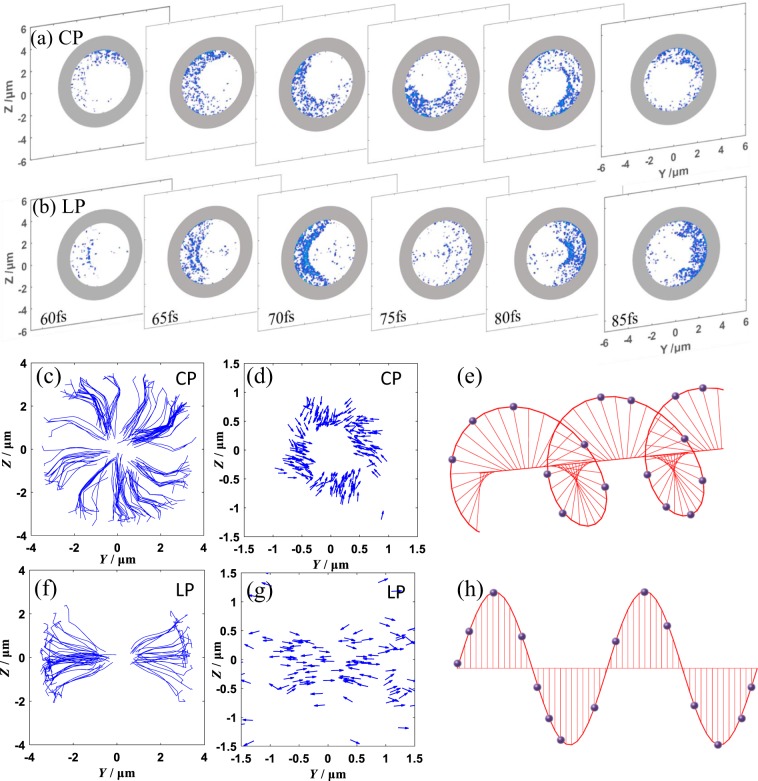
The density distribution of electrons at the middle of the channel respect to the time in the case of (**a**) the LP laser and (**b**) the CP laser. (**c**) Trajectories of high-energy electrons during 30 fs~125 fs. (**d**) The momentum distributions of high-energetic electrons at fixed time moment of 90 fs in the Y-Z plane and (**e**) the schematic diagrams of electron field corresponding to radiating CP laser. (**f**–**h**) respond to same figure captions with (**c**–**e**) in LP laser.

To see the AM transferring more clearly, we display the density of electrons within the laser pulse length in Fig. 3. The electron density distribution on the y-z plane at the middle of the channel is shown at the time interval of 5 fs in Fig. 3(a). Symmetric pattern about the z = 0 axis can be found for the LP laser, however in the CP-laser case, a clear helical bunch is observed, matching the density distribution of photons shown in Fig. 1 very well. But from these alone, one cannot infer whether each of the electrons is circulating around the propagation axis or it is the collective effect of the whole electron beam. The electron trajectories and their momenta on the y-z plane are summarized in Fig. (c,f,d,g). In the LP laser case, the electrons are extracted directly towards the central axis of the channel, following the laser polarization direction, as shown in Fig. 3(f,g). Hence the beam does not contain any angular momentum.

The CP laser, as seen in Fig. 3(c,d), not only drags the electrons towards the central axis, but also induces a lateral motion for each of them. The interesting feature is that electrons circulate around the axis at first and then keep their transverse momentum directions almost constant (the tangent of the trajectory curve first varies significantly and then remains almost constant). Their momenta, however, do not point to exactly to the axis but with a displacement angle, as illustrated by the tangent of the trajectories in Fig. 3(c). We can conclude that single electron does not carry significant OAM (circulate by itself) and the value yp_z_-zp_y_ is finite and it will move along the direction of the Lorentz force. The OAM of the electron bunches come from the collective azimuthal momentum p_φ_ of all the electrons. While long-wavelength radiation is recorded on the grids in PIC simulations, the high photon-energy emissions are usually treated as point-like particles, therefore the photon state describing the topological charge cannot be resolved. The extremely high OAM from the simulations does not reflect a topologically charged photon state, but rather the collective bunch-OAM originating from the azimuthal momentum p_φ_ of all photons.

It is therefore important to know how the electrons gain lateral momentum in the CP laser field. Electrons in the channel are injected from the outer edge of the laser beam and accelerated to gain high momentum along the laser propagation direction. Due to the slightly smaller velocity of electrons as compared to the laser field, there is a phase delay between them. For LP lasers as shown in Fig. 3(h), electrons always experience axial electric force at any phase thus no azimuthal momentum p_φ_ exist. In the CP laser case, the electrons slip to a later phase, as shown in Fig. 3(e), the electric field orientation is varied from the one at an earlier moment. This change leads to azimuthal momentum around the axis.

## Sources of photon OAM

**Figure 4:**
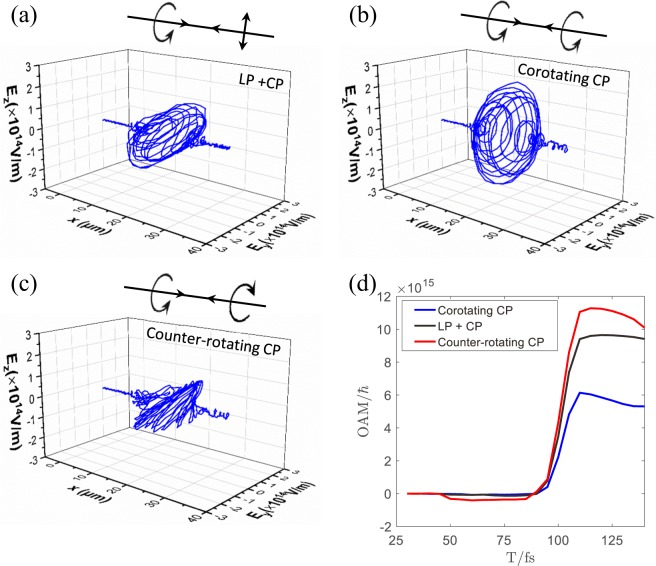
The electric field distributions along x axis responding to two counter-propagating laser pulses with (**a**) circular and linear polarization respectively (**b**) the corotating circularly polarization and **(c**) the counter-rotating circularly polarization. (**d**) The OAM of photons respect to the time in the three different situations mentioned above.

We notice that there are two possible sources of photon OAM from the ICS process. One is the reflected CP laser carrying SAM along the original propagation direction (+x axis), because the photon spin does not change its orientation from reflection. The other is the accelerated electrons pulled from the micro-channel with large OAM. To find out which one is the major source of the gamma-photon OAM, we remove the reflecting foil and let another laser collide with the electrons. The counter-propagating laser pulse is switched between LP (a = 20), left-hand rotation and right-hand rotation CP (a = 14). The electric field distributions along the x-axis when both laser pulses overlap are shown in Fig. 4(a–c). When the CP laser pulse from left encounter the LP laser pulse, simultaneously the LP laser interact with the electrons pulled from the micro-channel by the CP laser, as shown in Fig. 4(a) (overlapping of both lasers generate elliptically polarized laser field). The OAM of electrons increase with time obviously, indicating that electrons carry the OAM accelerated by the CP laser. These electrons interact with the LP laser which radiated from the right in the channel, producing OAM of the photons up to 9.48 × 10^15^ *ħ*, as shown in black line in Fig. 4(d).

In contrast, varying the LP laser to a CP laser which has the corotating laser field with the incident CP laser from left, these two laser pulses have opposite chirality and the laser amplitude is enhanced, as shown in Fig. 4(b). We can find that the OAM of photons elevated up to 1.14 × 10^15^ *ħ* as shown in Fig. 4(d) (red line). The additional OAM of photons originate from the incident laser from the right. We then change the colliding CP laser to have counter-rotating laser field with the driving CP laser. Overlapping of the two pulses produces a LP laser filed, as shown in Fig. 4(c). The OAM of the photons decreases to 5.69 × 10^10^ *ħ*. What we can see from this comparison is that about half of the OAM for the gamma photons come from the electrons and half from the scattering laser. In our single-pulse scheme, the picture is close to Fig. 4(a). The reflected laser is depleted during propagation therefore the field strength is marginally smaller as compared to that in Fig. 4(a). Accordingly, the peak OAM of gamma-photons in Fig. 2(d) is lower.

## Conclusions

In conclusion, the emission of *γ*-Ray and the transferring process of OAM in laser-driven MCP targets, based on the inverse Compton-scatting, has been studied though 3D PIC simulations and theoretical analysis. As a CP laser enters the channel, electrons located in the skin layer of the channel are extracted into the channel. These electrons with proper phase are accelerated and gaining OAM from the SAM of CP laser. Employing the colliding geometry results in the emission of *γ* rays with OAM.

## Methods

Simulations in this article were performed with the full 3D PIC code EPOCH^35,36^. EPOCH is a code with the standard relativistic electromagnetic (1D-3D) particle-in-cell algorithm, which implements a Monte Carlo algorithm to describe gamma-ray photon emission and pair production. The size of the simulation box is 70λ_0_ × 30λ_0_ × 30λ_0_ (λ_0_ = 0.8 μm is the central wavelength of the incident laser pulse) in the x × y × z directions, respectively, divided into 900 × 350 × 350 cells. Each cell was filled with 9 macro-particles. A Gaussian laser pulse with a FWHM duration of 30 fs and FWHM focal spot diameter of 6 μm was used, resulting in a = 14 for the circularly-polarized (CP) laser and a = 20 for the linearly-polarized (LP) laser. Here *a* = *eE*_*L*_/*m*_*e*_*ω*_0_*c* is the normalized laser amplitude, e and m_e_ are the electron charge and mass, E_L_ the laser electric field, ω_0_ the laser frequency, and c the speed of light in vacuum, respectively. As sketched in Fig. 1(a), we employ a carbon micro-channel target with inner diameter of 6 μm and electron density of n_e_ = 50 n_c_ (n_c_ = m_e_ε_0_ω^2^/e^2^). The channel has an outer diameter of 8 μm and varying length. To enable laser-electron collision, we attach a flat foil to the rear side of the structure, of thickness 5 μm and electron density n_e_ = 50 n_c_. The plasma target is initially cold. In our simulations, we focus on electrons and photons moving in the forward direction (along the +x axis).
